# Regenerative Endodontic Procedure (REP) of a Previously Treated, Infected, Immature Permanent Incisor

**DOI:** 10.1155/crid/8600738

**Published:** 2026-07-08

**Authors:** Heather T. Morris, Patrick Timothy Ruck

**Affiliations:** ^1^ Department of Pediatrics, University of Cincinnati College of Medicine, and Division of Pediatric Dentistry, Cincinnati Children′s Hospital Medical Center, Cincinnati, Ohio, USA, uc.edu

**Keywords:** mineral trioxide aggregate, regenerative endodontics, root canal preparation, root canal therapy

## Abstract

The management of immature permanent teeth with pulpal necrosis and prior endodontic failure remains clinically challenging due to incomplete root development, persistent infection, and reduced structural integrity. Regenerative endodontic procedures (REPs) offer a biologically driven alternative to apexification; however, their use in retreatment scenarios is not well documented. This report describes the successful treatment of a previously treated immature maxillary central incisor in a 7‐year‐old patient presenting with a chronic apical abscess and a longstanding sinus tract. Prior endodontic therapy was inadequate, with insufficient obturation and a poorly adapted full‐coverage restoration contributing to coronal leakage and reinfection. Management involved removal of the existing root filling material, limited mechanical instrumentation, chemical disinfection with low‐concentration sodium hypochlorite, placement of a triple‐antibiotic paste, and subsequent induction of intracanal bleeding. Mineral trioxide aggregate was placed over the resulting scaffold, followed by definitive coronal restoration. Clinical and radiographic evaluations at 3, 12, and 34 months demonstrated complete resolution of apical pathology, absence of clinical symptoms, increased dentinal wall thickness, and continued root maturation with apical closure. This case supports the potential application of regenerative endodontic therapy in previously treated immature teeth with persistent infection. Establishing conditions favorable to biologic healing may allow preservation of regenerative capacity even in retreatment contexts. Further investigation is needed to clarify indications, predictability, and long‐term outcomes of REPs in similar cases.

## 1. Introduction

Necrosis of immature permanent incisors poses a substantial clinical challenge in endodontic practice, primarily because normal root maturation is disrupted following pulpal vitality loss [1]. As a result, affected teeth frequently exhibit underdeveloped roots characterized by thin dentinal walls, reduced root length, and enlarged canal spaces with open apices, all of which contribute to structural fragility and increased susceptibility to fracture [2, 3].

Traditional management of these cases has relied on apexification procedures, most commonly using calcium hydroxide or calcium silicate–based materials to establish an apical barrier that permits subsequent obturation [3]. Although such approaches can facilitate root canal treatment, they do not promote continued root development or reinforcement of dentinal walls. Additionally, extended calcium hydroxide therapy has been associated with an elevated risk of root fracture over time [4]. Mineral trioxide aggregate (MTA) apexification offers a more predictable apical seal and may allow limited apical closure; however, treated teeth often retain unfavorable structural characteristics, including thin radicular dentin and compromised crown‐to‐root ratios, which may predispose them to cervical fracture [5]. These limitations have led to increasing interest in biologically based treatment approaches aimed at promoting continued root maturation rather than merely creating an apical stop [6].

Regenerative endodontic procedures (REPs), as defined by the American Association of Endodontists, are designed to restore functional tissue within the root canal system through biologically driven processes that support the regeneration or repair of the pulp–dentin complex [7, 8]. Immature teeth are particularly well‐suited for these procedures due to the presence of stem cells and a rich vascular supply in the apical region, which together provide the biologic foundation necessary for tissue regeneration [9]. Evidence from both experimental and clinical studies, particularly involving replanted immature teeth, has demonstrated that revascularization of necrotic pulps is achievable when appropriate conditions are established [10, 11].

Successful regenerative outcomes depend on establishing an environments conducive to cell migration, proliferation, and differentiation. A disinfected canal space, even if necrotic, may serve as a pathway for the ingrowth of cells originating from the apical tissues [12]. However, persistent microbial contamination can impede this process, underscoring the importance of effective canal disinfection before regenerative attempts [13–15]. Current protocols emphasize chemical debridement with low‐concentration sodium hypochlorite, minimal or no mechanical instrumentation, and the use of intracanal medicaments, such as calcium hydroxide or antibiotic pastes, to reduce bacterial load [7, 8]. Following disinfection and resolution of clinical symptoms, induction of intracanal bleeding creates a biologic scaffold that facilitates the recruitment and differentiation of stem cells necessary for tissue formation [7, 8]. Clinical reports have demonstrated that this approach can result in continued root development, including increased root length, thickening of dentinal walls, and eventual apical closure [16]. Moreover, REPs have shown promise in managing complex clinical situations, including cases involving root fractures and external resorptive defects [6].

Despite these advances, there remain limited to no case reports regarding the use of REPs in immature teeth that have previously undergone unsuccessful nonsurgical root canal therapy, particularly in the presence of long‐standing infection or sinus tract formation. The capacity of such teeth to achieve continued root maturation under regenerative conditions is not yet well defined [17].

The purpose of this report is to present the successful management of a previously treated, infected, immature permanent incisor using a regenerative endodontic approach.

## 2. Case Description

**Figure 1:**
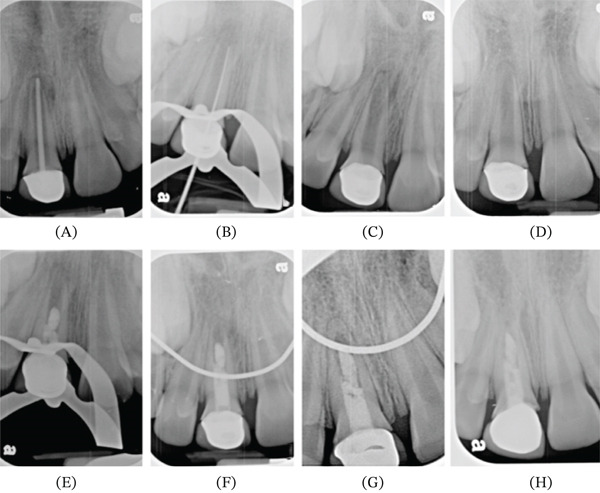
The right central permanent incisor treatment history and demonstration of apical development. (A) Preoperative radiograph revealing the maxillary right central incisor with an open apex and underfilled root canal obturation with gutta percha, a PFM crown, and recurrent abscess. (B) Intraoperative radiograph showing removal of gutta percha, open apex, and estimated working length. (C) Periapical radiograph taken status post 2 months of antibiotic therapy. (D) Periapical radiograph taken status post 3 months of antibiotic therapy E. Periapical radiograph taken demonstrating the placement of an MTA barrier. (F) Periapical 3‐month follow‐up radiograph showing healing of periapical defect. (G) Twelve‐month follow‐up radiograph showing resolution of periapical defect and hard tissue deposition at root apex. (H) Thirty‐four‐month post‐REP showing radiographic resolution of periapical abscess and apical maturation. PRICE 2020 flowchart presenting the steps involved in the case report (adapted from Nagendrababu et al., 2020).

A 7‐year‐old black male was seen in a hospital pediatric dental clinic for a recurring abscess on his permanent maxillary right central incisor. His social and medical history were noncontributory. An in‐depth dental history with the patient′s mother revealed that he had suffered a complicated crown fracture 1 year earlier while playing with his brother. The patient was seen by an outside private practice dental office for treatment. The tooth was treated via conventional nonsurgical root canal therapy due to pulpal necrosis as a sequela of dental trauma. Because of substantial clinical crown loss, the tooth was then restored with a porcelainfused‐to‐metal (PFM) crown (Figure 1A). It should be highlighted that the endodontic therapy provided to the patient did not appear to meet clinical standards [18]. The tooth appeared underfilled, with the previous provider not addressing the unique challenges that immature permanent teeth pose due to their wider canal spaces and developing apical tissues [19]. In addition, the existing PFM crown did not have adequately sealed margins and was not an appropriate restorative choice for the patient′s and tooth development [20, 21]. The inadequate endodontic therapy in conjunction with a leaking PFM restoration most likely contributed to bacterial invasion and treatment failure [22–24].

At the dental exam, the patient′s mother reported a persistent, localized gingival swelling apical to the treated tooth. The patient had been seen at the same outside office additional times post root‐canal therapy without resolution of the gingival swelling. At exam, the patient denied a history of extraoral edema, fever, or pain, but had experienced periods of localized gingival edema for over a year at the apical area of the maxillary right central incisor. Clinical exam revealed tenderness to percussion and palpation, a sinus tract, and Class I mobility. The PFM crown was found to have open margins. Radiographic exam showed the apex of the maxillary right central incisor to be open 2.5 mm with a periapical lesion measuring 8.5 × 3.5 mm. The tooth was diagnosed as a previously endodontically treated, with a chronic apical abscess. Treatment options were presented to the patient′s parent, including retreatment with a REP, apexification with a bioceramic material, or extraction and replacement with a prosthesis. The parent and patient consented to the REP.

On the next visit, the tooth was anesthetized and isolated with a rubber dam. Access was obtained through the existing crown, and the gutta‐percha was removed (Figure 1B). The canal was not shaped, but was cleaned and instrumented with a broach and K‐file instrument, and then copiously and gently irrigated with a total of 20 mL of 1.5% sodium hypochlorite (NaOCl, Vista Apex, Racine, Wisconsin, United States) using a 3 mL side‐vented syringe. Lower concentrations of NaOCl (20 mL/canal, 5 min) are advised to minimize cytotoxicity to potential stem cells in the apical tissues [25]. It was then rinsed with 17% EDTA Vista Apex, Racine, Wisconsin, United States) (20 mL/canal, 5 min) and dried with sterile paper points [25]. A 1:1:1 creamy mixture of ciprofloxacin (125 mg), metronidazole (125 mg), and minocycline (200 mg) was compounded using sterile water and spun into the canal using a Lentuo spiral in a slow‐speed handpiece. Per AAE guidelines, the triple antibiotic paste was mixed to a final concentration of 1–5 mg/mL [25]. The access was closed with a sterile cotton pellet and temporary restorative material (Cavit, 3M ESPE, St Paul, Minnesota). An additional periapical radiograph was taken at a subsequent visit to monitor healing (Figure 1C).

After 3 months of antibiotic disinfection and confirmation of symptom resolution, the patient returned for continuation of the REP. There was no sinus tract noted at this visit (Figure 1D). After achieving local anesthesia with 3% mepivacaine without vasoconstrictor and isolating with a rubber dam, the temporary restoration and cotton pellet were removed. The canal was flushed with a gentle, copious irrigation of 20 mL of 17% ethylenediaminetetraacetic acid using a side vented syringe (EDTA, Vista Apex, Racine, Wisconsin, United States), and the canals were then dried with paper points [25]. A sterile Hedstrom file was used to agitate 2 mm past the apical foramen to induce bleeding. Blood was allowed to fill the canal and clot over 10 min. White ProRoot MTA (Dentsply Sirona, Charlotte, North Carolina, United States) was placed in the canal (Figure 1E) over the blood clot as a capping agent. GI (Fuji IX, GC America, Alsip, Illinois, United States) was placed over the MTA capping material, and a composite restoration (TPH LV A1, Dentsply Sirona, Charlotte, North Carolina, United States) was placed in the access. On subsequent PA film, a larger amount of MTA was noted, more apically placed than originally planned (Figure 1F). This most likely occurred because no collagen‐resorbable matrix was placed on top of the blood clot to guide it. The patient also received a Nance space maintainer at this visit. The patient′s mother was informed that the ideal treatment for the tooth would be to place a new, better‐fitting, esthetic, full‐coverage restoration in the form of a composite strip crown to allow for continued craniofacial growth and development [20, 21].

The patient presented 3 months after MTA placement for a follow‐up exam and radiographs. The patient denied any history of pain or swelling associated with the permanent maxillary right central incisor. Clinically, the patient was negative to percussion and palpation, with no mobility noted. No gingival irritation, erythema, edema, or sinus tract was observed. A periapical radiograph showed no signs of periapical pathology (Figure 1G). Follow‐up exams were performed at 3 months (Figure 1F), 12 months (Figure 1G), and 34 months (Figure 1H) post‐regenerative procedure. The tooth continued to be symptom‐free. Periapical radiographs showed healing of the alveolar defect, thickening of the roots, and gradual deposition of hard tissue at the root apex (i.e., apical closure). After 34 months, the patient did not return for any further follow‐up appointment. At each follow‐up, the mother was instructed that replacement of the PFM crown on the permanent maxillary right central incisor should still be considered.

## 3. Discussion

Management of immature permanent teeth is inherently complex because root formation remains incomplete at the time of treatment. The combination of wide canal space, thin radicular dentin, and open apices complicates both instrumentation and long‐term structural stability of these teeth [26]. These anatomic features increase the risk of procedural complications, such as inadequate debridement, overextension of materials, and iatrogenic damage. In addition, restorative planning must account for the ongoing development of the tooth and its long‐term functional demands. The present case illustrates many of these challenges, particularly in the context of prior inadequate treatment.

Root development begins once enamel and dentin formation reach the region of the future cementoenamel junction, after which Hertwig′s epithelial root sheath (HERS) extends apically and directs root morphogenesis [27, 28]. As dentin deposition progresses, HERS undergoes fragmentation, leaving epithelial remnants within the periodontal ligament while continuing to influence root patterning and odontoblast differentiation [27–29]. At the apical level, the epithelial diaphragm contributes to shaping the apical foramen [28]. In the setting of trauma or infection, HERS can be disrupted; however, the biologic activity of the apical tissues may still permit continued root development under favorable conditions [30]. Preservation of this apical environment is critical because it supports the differentiation of mesenchymal stem cells into odontoblast‐like cells capable of producing hard tissue [28, 30]. When HERS integrity is compromised, alternative mineralized tissues, such as cementum‐like or bone‐like structures, may form in place of normal dentin [30].

Histologic and clinical evidence has demonstrated that REPs can lead to increases in root length, narrowing of the apical diameter, and thickening of canal walls [31]. That said, the tissues formed following REPs are frequently characterized as reparative rather than fully regenerative, often consisting of a mixture of cementum‐like, bone‐like, and periodontal ligament–like tissues rather than a true pulp–dentin complex [31, 32]. Partial regeneration has been described in some studies, with findings that include fibrovascular connective tissue, odontoblast‐like cells, and markers suggestive of vascular and neural elements [31, 32]. These observations indicate that outcomes following REPs exist along a spectrum, influenced by local biologic conditions and the extent of tissue preservation.

Control of microbial infection is widely regarded as a determining factor in regenerative success. Persistent intracanal bacteria are strongly associated with treatment failure, particularly in previously treated teeth [33]. Although complete sterilization of the canal system is unlikely, reducing the bacterial load to a level compatible with healing appears sufficient for tissue ingrowth. Interestingly, evidence suggests that root maturation can occur even when some microorganisms remain, provided that apical tissues, such as HERS and the apical papilla, retain their functional capacity [30, 34]. This suggests that biologic viability of key tissues, in some cases, may outweigh the need for absolute disinfection.

Root development depends on coordinated interactions between epithelial and mesenchymal components, particularly HERS cells and odontoblast‐lineage cells derived from stem cells [28]. Stem cells from the apical papilla (SCAP) are especially important because of their high proliferative potential and their ability to remain viable even in inflamed environments [34, 35]. In addition to SCAP, other stem cell populations, including those from dental pulp, periodontal ligament, and bone marrow, may contribute to tissue formation within the canal space [35]. These cell populations provide the biologic basis for continued root maturation following regenerative procedures.

Following adequate disinfection, induction of bleeding into the canal space introduces a scaffold rich in cells and signaling molecules that support tissue formation [26, 35]. Within this environment, SCAP and other progenitor cells can migrate, proliferate, and differentiate, contributing to continued root development [26, 35]. The biomaterials used in these procedures further influence outcomes. Calcium silicate–based materials, such as MTA, promote cell differentiation, mineral deposition, and the release of signaling molecules, including growth factors such as transforming growth factor beta, which support tissue repair and regeneration [36]. Their sealing ability also helps maintain a stable environment limiting reinfection and supporting healing [36].

From a microbiologic standpoint, differences between primary and persistent infections are particularly relevant in retreatment cases. Initial infections are typically dominated by anaerobic Gram‐negative organisms such as *Porphyromonas*, *Prevotella*, and *Fusobacterium* [22]. In contrast, teeth requiring retreatment are more frequently associated with Gram‐positive facultative species, especially *Enterococcus faecalis*, which exhibit enhanced survival mechanisms [22–24]. These organisms are capable of resisting antimicrobial agents, tolerating alkaline environments, and penetrating dentinal tubules, making them difficult to eliminate [22–24]. In addition, the presence of biofilms further protects bacterial communities from both host defenses and intracanal medicaments [23].

For this reason, endodontic failure is typically multifactorial, involving persistent infection, inadequate chemomechanical preparation, and coronal leakage [23]. Even with appropriate techniques, microorganisms may persist within anatomical complexities such as lateral canals and dentinal tubules [22, 23]. Clinical studies of retreatment cases consistently demonstrate that although bacterial levels can be significantly reduced, complete elimination is rarely achieved [24]. This reinforces the concept that treatment success depends not on sterility, but on creating conditions that allow host‐mediated healing.

In the present case, the favorable outcome observed, despite prior inadequate treatment and prolonged infection, may be attributed to effective reduction of microbial load, preservation of apical tissues, and establishment of a biologically supportive environment. Collectively, these factors likely enabled continued root maturation and resolution of periapical pathology.

This report is limited by its single case design, which restricts generalizability. Additionally, histologic confirmation of the tissue type formed was not available, and outcomes are based on clinical and radiographic findings. Despite these limitations, the extended follow‐up period and consistent findings provide meaningful insight into the potential application of regenerative techniques in retreatment scenarios.

## 4. Conclusion

This case highlights the potential of REPs as a treatment option for immature permanent teeth with failed prior endodontic therapy and persistent infection. Despite suboptimal initial management and prolonged pathology, favorable clinical and radiographic outcomes were achieved, including resolution of periapical disease and continued root development.

These findings suggest that the regenerative capacity of immature teeth may persist even after treatment failure when a biologically supportive environment is re‐established through appropriate disinfection, scaffold formation, and preservation of apical tissues.

Although limited by the nature of a single case report, this study supports consideration of regenerative approaches as an alternative to apexification or extraction in retreatment scenarios. Additional clinical studies are needed to define prognosis better, refine treatment protocols, and evaluate long‐term outcomes.

## Author Contributions

**Heather T. Morris:** conceptualization, investigation, methodology, writing ‐ original draft, writing – review and editing. **Patrick Timothy Ruck:** investigation, methodology, writing – original draft, writing – review and editing.

## Funding

No funding was received for this manuscript.

## Disclosure

All authors have read and approved the final version of the manuscript. Dr. Patrick Timothy Ruck and Dr. Heather T. Morris had full access to all of the data in this study and takes complete responsibility for the integrity of the data and the accuracy of the data analysis.

## Ethics Statement

The study is not considered human research and is therefore exempt.

## Conflicts of Interest

The authors declare no conflicts of interest.

## Data Availability

The data that support the findings of this study are available from the corresponding author upon reasonable request.
